# Cloning and physical localization of male-biased repetitive DNA sequences in *Spinacia oleracea* (Amaranthaceae)

**DOI:** 10.3897/CompCytogen.v15i2.63061

**Published:** 2021-04-23

**Authors:** Jian Zhou, Shaojing Wang, Li’ang Yu, Ning Li, Shufen Li, Yulan Zhang, Ruiyun Qin, Wujun Gao, Chuanliang Deng

**Affiliations:** 1 College of Life Sciences, Henan Normal University, Xinxiang 453007, China Henan Normal University Xinxiang China; 2 Department of Plant Biology, University of Illinois at Urbana-Champaign, Urbana, IL 61801, USA University of Illinois at Urbana-Champaign Urbana United States of America

**Keywords:** FISH, Genomic subtraction hybridization, Retrotransposon, Sex chromosome evolution, Spinach

## Abstract

Spinach (*Spinacia oleracea* Linnaeus, 1753) is an ideal material for studying molecular mechanisms of early-stage sex chromosome evolution in dioecious plants. Degenerate oligonucleotide-primed polymerase chain reaction (DOP-PCR) technique facilitates the retrotransposon-relevant studies by enriching specific repetitive DNA sequences from a micro-dissected single chromosome. We conducted genomic subtractive hybridization to screen sex-biased DNA sequences by using the DOP-PCR amplification products of micro-dissected spinach Y chromosome. The screening yielded 55 male-biased DNA sequences with 30 576 bp in length, of which, 32 DNA sequences (12 049 bp) contained repeat DNA sequences, including *LTR/Copia*, *LTR/Gypsy*, simple repeats, and DNA/CMC-EnSpm. Among these repetitive DNA sequences, four DNA sequences that contained a fragment of *Ty3-gypsy* retrotransposons (SP73, SP75, SP76, and SP77) were selected as fluorescence probes to hybridization on male and female spinach karyotypes. Fluorescence *in situ* hybridization (FISH) signals of SP73 and SP75 were captured mostly on the centromeres and their surrounding area for each homolog. Hybridization signals primarily appeared near the putative centromeres for each homologous chromosome pair by using SP76 and SP77 probes for FISH, and sporadic signals existed on the long arms. Results can be served as a basis to study the function of repetitive DNA sequences in sex chromosome evolution in spinach.

## Introduction

Sex chromosomes evolved from autosomes by stages; the key event in the evolution of sex chromosomes includes the emergence of sex-determining genes, recombination suppression, accumulation of repetitive sequences, degeneration of Y chromosome, and dosage compensation effect of X chromosome (Abbott et al. 2017). Sex chromosomes in mammals are mostly ancient, but sex chromosomes in plants, insects and some fishes have recently evolved. In all evolutionary steps, recombinant suppression is a key step in the evolution of sex chromosomes. The ceasing of recombination avoids the occurrence of progeny sterilization or hermaphroditism among progenies. In early-stage sex chromosome evolution, the recombination of male-specific regions on the Y chromosome is reduced or restricted (Akagi et al. 2014). The size of the recombination suppression region and differences of size between X/Y or Z/W chromosomes generally reflect the stage of sex chromosome evolution. Homomorphic chromosomes are generally considered to be relatively young sex chromosomes, and their non-recombination regions are commonly very small. For example, asparagus (*Asparagus officinalis* Linnaeus, 1753) has a very young pair of sex chromosomes and very small male-specific regions (Harkess et al. 2017). Papaya (*Carica papaya* Linnaeus, 1753) also has homomorphic sex chromosomes with larger sex-specific regions than asparagus. Analysis of high-density linkage map of papaya revealed that 225 out of the 347 markers co-segregated with sex phenotype. This finding revealed the severe recombination suppression around the sex-determining site (Ma et al. 2004). Spinach (*S. oleracea* L., 2n = 12) is a diploid dioecious leafy vegetable with a pair of homomorphic sex chromosomes (Arumuganathan et al. 1991; Xu et al. 2017). In spinach, a Y-chromosomal region around the male-determining locus does not recombine with the counterpart region on the X chromosome (Takahata et al. 2016; Kudoh et al. 2018).

Repetitive DNA sequences, primarily transposons, retrotransposons (RTs), and tandem repeats (satellite DNA, small satellite DNA, and microsatellite DNA sequences), make up the majority of all the nuclear DNA in most eukaryotic genomes (Biscotti et al. 2015). These sequences used to be called garbage sequences, but their substantial roles in a variety of biological processes, including gene expression, transcriptional regulation, chromosome structure construction, have been recently discovered. Their functions and evolution are popular research topics (Mehrotra et al. 2014; Cioffi et al. 2015). Accumulation of repetitive sequences is among the most common features of the sex chromosomes of dioecious species. A great portion of repetitive sequences has been identified from the sex chromosomes of humans (Erlandsson et al. 2000), mammals (Dechaud et al. 2019), fish (Faber-Hammond et al. 2015), birds (Zhou et al. 2014), and insects (Bachtrog et al. 2003). In flowering plants, highly repetitive regions were distributed on the sex chromosomes of dioecious plants with heteromorphic sex chromosomes, such as *Silene latifolia* Poire, 1789 and *Rumex acetosa* (Linnaeus, 1753) (Hobza et al. 2017), similar to some dioecious plants with younger sex chromosomes. Papaya is a model species for studying early-stage sex chromosomes (Ming et al. 2008). The Y chromosome contains a small male-specific (MSY, 8.1 Mb, only 13% of the entire Y chromosome) (Vanburen et al. 2015). Studies have shown the enriched repetitive DNA sequences in this region (Wang et al. 2012). Further studies showed that repetitive DNA sequences accounted for approximately 77% of hermaphrodite-specific region in the Y chromosome (HSY), 79.2% of MSY, and 67.2% on the X-counterpart, the values of which were significantly higher than the ratio of repeat sequences in the entire genome (51%) (Yu et al. 2007; Na et al. 2014). The types of transposons in the HSY region, including *Ty1*-*Copia* and *Ty3*-*Gypsy*, are primarily RTs. The expansion of the sex-determining region was proposed to be related to the accumulation of *Ty3-gypsy*RTs (Na et al. 2014). BAC sequencing revealed that the Y-chromosome region around the male-determining locus in spinach contains a large amount of repetitive elements, most of which are novel *Ty1-copia-like* and its derivative elements (Kudoh et al. 2018). However, the BAC clone sequences account for only a part of the sex-linked non-recombining region. Further experiments are needed to determine the size of the sex-linked region in spinach.

Genomic subtraction is used for isolating DNA that is absent in deletion mutants. The method removes the sequences present in the wild-type (tester DNA) and the deletion mutant genomes (driver DNA) from wild-type DNA featured by simple, rapid, sensitive, and economic means. This technique is widely applied in the separation and identification of gene rearrangement and in the preparation of polymorphism loci probe (Straus et al. 1990; Hou et al. 1995; Asalone et al. 2019). However, whether the technique can be used to screen sex chromosome-specific DNA sequences has not been reported.

In this study, the X and Y chromosome of spinach were successfully isolated and amplified by degenerated-oligonucleotide-primed polymerase chain reaction (DOP-PCR) (Deng et al. 2013). Single chromosome DOP-PCR amplified products tend to enrich chromosome-specific DNA repeat sequences (Zhou et al. 2007). Then, using X chromosome DOP-PCR amplified products as the driver DNA and Y chromosome DOP-PCR amplified products as the tester DNA, the enriched repetitive DNA sequences on the Y chromosome were obtained by genomic subtraction hybridization. Our study provided a new approach for exploring enriched DNA repetitive sequences from spinach Y chromosome.

## Material and methods

### Plant materials

The seeds of spinach (*S. oleracea* Linnaeus, 1753, cv. Japan) were planted in the garden field of Henan Normal University under natural conditions. Genomic DNA from each male and female spinach was extracted from young leaves using the traditional cetyltrimethylammonium bromide method (Rogers et al. 1989).

### Microdissection of X and Y chromosome in spinach

The X/Y chromosome is the largest submetacentric chromosome (Ellis et al. 1960; Deng et al. 2013). The microdissection of the largest chromosome in spinach was carried out according to the procedures described by Deng et al. (2013). Initially, the largest chromosome in spinach was identified based on its size and was microdissected using a glass needle that was fixed to the arm of a Leitz micro-operation instrument on an inverted phase-contrast microscope (Olympus 1 M, Japan). Ten chromosomes were isolated. The microdissected chromosome was collected into an Eppendorf tube (0.2 mL) and separately digested with proteinase K buffer at 0.5 mg/mL (Roche, Indianapolis) in 1× Taq polymerase buffer (Promega, Madison). The isolated chromosomal DNA was incubated in a proteinase K solution at 37 °C for 2 h. Proteinase K was then inactivated at 90 °C for 10 min. Then, the chromosome DNA was amplified by DOP-PCR in a PTC-200 thermocycler (MJ Research, Watertown, MA, USA). To obtain high-concentration DNA products for genomic subtraction library construction, the DOP-PCR products were amplified from X and Y chromosome by two rounds of recursive enrichment based on previous studies (primer sequence: CCGACTCGAGNNNNNNATGTGG) (Deng et al. 2013).

### Construction of Y chromosome genomic subtraction library

The modified DOP-PCR primer that contains *Bam*H I digestion site (modified primer sequence: CGGAGGATCCNNNNNNATGTGG), was used to amplify the products from the second round spinach Y chromosome DOP-PCR amplification. DOP-PCR amplification was performed in 50 μL reaction volume containing 1 × PCR buffer, 1.5 mmol/L dNTP Mixture (Transgene, Beijing, China), 2.5 U *Taq* polymerase (Takara, Bejing, China), 100 ng template DNA, and 0.2 μM primer. The amplification was performed by initial denaturation at 94 °C for 5 min, followed by 30 cycles of denaturation at 94 °C for 1 min, annealing temperature 55 °C for 90 s, extension at 72 °C for 3 min, and a final extension at 72 °C for 10 min. Then, the concentration of the amplified DOP-PCR products was quantified, followed by enzyme digestion in 25.0 μL volume containing 2 × K buffer and 20 000 U *Bam*H I (Takara, Beijing, China) at 37 °C for 3 h in a metal bath for further quantification and quality control.

As described from Y chromosome amplification, the modified DOP-PCR primer was used to amplify the second round DOP-PCR amplification products of spinach X chromosome along with the quality control and quantification of the products. Finally, the amplified products from both X and Y chromosomes were purified using Takara MiniBEST DNA Fragment Purification Kit Ver.4.0 (Takara, Beijing, China).

The DOP-PCR amplified products from the libraries of X (Driver DNA) and Y (Tester DNA) chromosomes were mixed in a 100:1 ratio for subsequent hybridizations. The mixed DOP-PCR amplified products were treated by water bath at 99 °C for 10 min, mixed with 4 mL of PERT (8% phenol, 1.25 M sodium perchlorate, and 0.12 M disodium hydrogen phosphate dissolved in 1000 mL of distilled water) for 72 h, annealed at 25 °C and at 100 rpm on the shaking table for 72 h (shaking for 8 h and stopping for 8 h), and then placed on the shaking table overnight. After 72 h of annealing, the hybridization solution was purified by suction filtration with a syringe and an organic filter. It was extracted twice with chloroform:isoamyl alcohol at 24:1, centrifuged at 12 000 rpm for 5 min, precipitated by 1% volume of sodium acetate and 2.5 volumes of absolute ethanol at -20 °C overnight, centrifuged at 10 000 rpm for 10 min, washed twice with 70% absolute ethanol, dissolved in 800 μL sterile ddH_2_O, and transferred into a 1.5 mL centrifuge tube. Then, the mass of the hybridization solution was quantified by microspectrophotometer for further steps.

Enzyme digestion, purification, dephosphorylation, and re-purification of the vector were conducted.

A mixture of 1.0 μL of PUC119, 2.0 μL of 10 × K buffer, 2.0 μL of *BamH* I, and 15 μL ddH_2_O were quantified into 20.0 μL for 3 h digestion in a 30 °C water bath. Then, purification was performed according to Takara MiniBEST DNA Fragment Purification Kit Ver. 4.0 (Takara, Beijing, China). The reaction mixture was placed in a 0.2 mL centrifuge tube containing 40.0 μL (1–20 pmol) vector DNA, 5.0 μL 10 × K alkaline phosphatase buffer, and 1.0 μL CIAP, and adjusted to 50 μL. The reaction was conducted in a metal bath at 37 °C for 15 min, and then at 50 °C for 15 min for dephosphorylation, purification, and mass quantification.

### Cloning of DNA subtraction library

Gradient design was carried out according to the ratio between the hybrid liquid and the vector, after which the optimized reaction mixture was placed in the microcentrifuge tube containing 1.5 μL PUC119 (Takara Code: 3319), 0.1 µL T4 DNA ligase (Takara Code: 2011A), and 2.0 µL 10 × buffer at contents up to 20 µL. The reaction was conducted in the metal bath at 16 °C for 5 h. The ligation products were transformed into competent cells, screened according to blue and white spots, and amplified by colony PCR using universal primer M13 (CGCCAGGGTTTTCCCAGTCACGAC).

### Screening and identification of DNA subtractive library

The selected recombinant plasmids were identified using the spinach female and male genomic DNAs as probes labeled with DIG (Roche: 11277065910) by dot hybridization method. Basically, the subtractive DNA libraries with male-specific DNA sequences were hybridized and formed colonies on films. These colonies were selected for further Sanger sequencing.

### Screening of repetitive DNA sequences

On the basis of the results of dot-blot hybridization, the male-hybridized colonies (PCR-amplified products derived from bacterial solution with more than 250 bp) were selected for Sanger sequencing at Shanghai Invitrogen Biotechnology Co., Ltd. The sequencing results were analyzed by BLASTn and RepeatMasker (http://www.repeatmasker.org/). Initially, sequencing products were blasted against the spinach reference genome (http://www.spinachbase.org/cgi-bin/spinach/index.cgi) with a cutoff of 90% similarity and E-value 1e-10 to prevent the contamination of the DNA from other organisms. Sequences with no hits were deleted. Then, the DNA sequences were aligned to RepeatMask libraries to classify the type of repeats. Ultimately, the DNA sequences were annotated using BLASTn against the NCBI nucleotide database. Based on the sequencing results, primers for each group of repetitive DNA sequences were designed by Oligo7 for PCR amplification (Suppl. material 1: Table S1). Amplification of those repetitive DNA was performed in a 20 μL reaction setting, which included 1 × PCR buffer, 0.75 mM dNTP Mixture, 1 U *Taq* polymerase (Takara, Bejing, China), 100 ng template DNA, and 0.1 μM primer. The reaction was carried out using the following cycle conditions: initial denaturation at 94 °C for 5 min, followed by 35 cycles of denaturation at 94 °C for 30 s, annealing temperature 55 °C for 45 s, extension at 72 °C for 1 min, and a final extension at 72 °C for 10 min. PCR products with a single amplified band were purified for fluorescent probe labeling.

### Chromosome localization of repeat DNA sequences

The spinach seeds were initially soaked in a moisturized and low-temperature (4 °C) environment overnight. Then, the seeds were placed in a constant temperature incubator at 25 °C in the dark. The seeds with approximate 1 cm root length were placed in a 1.5 mL centrifugal tube for nitrous oxide pretreatment. Subsequently, the roots were fixed in 90% glacial acetic acid for 10 min and finally stored in the refrigerator at -20 °C in 70% ethanol. Each selected tissue was rinsed by distilled water for 10 min, after which it underwent dissection and digestion using a solution containing 1% pectolyase Y23 (Yakult Pharmaceutical, Tokyo) and 2% cellulose Onozuka R-10 (Yakult Pharmaceutical, Tokyo) for 1.5 h at 37 °C (one section per tube with 20 µL of the enzyme solution). The abovementioned treated root sections were carefully split into individual cells by using needles and by intensive vortexing at room temperature along with soaking in 100% ethanol. Furthermore, the cells were collected from the bottom of the tube by centrifugation and re-suspended in an acetic acid ethanol solution (9:1 dilution). Finally, the cell suspension was dropped onto glass slides in a box lined with wet paper towels for observation.

Then, 45S rDNA (the probe was donated by Fangpu Han, a researcher from the Institute of Genetics and Developmental Biology, Chinese Academy of Sciences) was labeled using Alexa fluor-488-dUTP (green), and the male-specific bands were labeled using Texas-red-dCTP (red) with the nick translation method based on previous protocols (Birchler et al. 2008). The labeled probes were placed in a refrigerator with light prevention at -20 °C.

Fluorescence *in situ* hybridization (FISH) between spinach chromosomes at metaphase and DNA probes derived from each repetitive sequence was performed according to the method described in previous studies (Gao et al. 2011). Selected chromosome plates during the metaphase-stage for hybridization were placed into the ultraviolet crosslinker for 2 min with 0.125 J. A probe solution containing in 2 × SSC and 1 × TE was added to the slides. After denaturation in boiling water for 5 min, the slides with probe were incubated at 55 °C in a humid chamber for 8–12 h. After hybridization, the slides were washed in 2 × SSC and mounted on Vectashield mounting medium containing 1.5 μg/ml 4ʹ,6-diamidino-2-phenylindole (Vector Laboratories, Burlingame, USA). The FISH images were captured with an ANDOR CCD under an Olympus BX63 fluorescence microscope. The images were processed by Adobe Photoshop 7.0.

## Results

### Preparation of Tester DNA and Driver DNA

**Figure 1.**
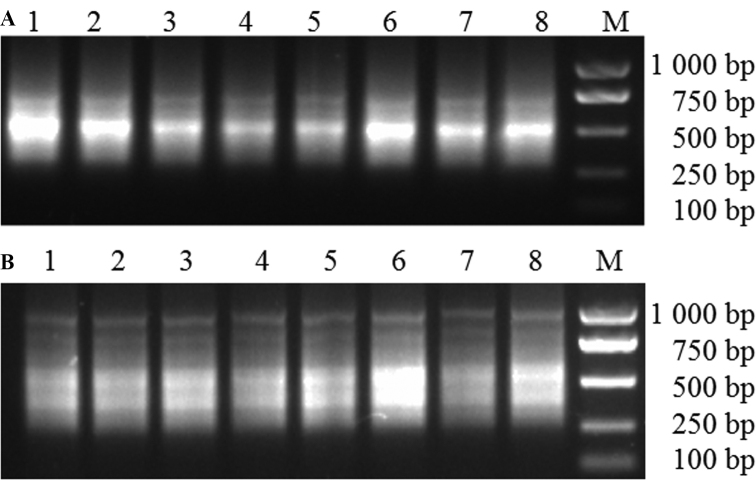
Gel electrophoresis results of spinach Y (**A**) /X (**B**) chromosome DOP-PCR products using modified primer Lane M: Trans 2K plus DNA Marker; Lane 1–8(**A**): spinach Y chromosome second round DOP-PCR products as template using modified primer; Lane 1–8(**B**): spinach X chromosome second round DOP-PCR products as template using modified primer

The amplified products from the X/Y chromosome were identified using a male-specific marker T11A (Onodera et al. 2011). Two X chromosomes and three Y chromosomes were successfully microdissected and amplified (Suppl. material 1: Fig. S1). The major Y chromosome DNA products amplified from DOP-PCR ranged from 200 bp to 1500 bp, and the DNA products of around 500 bp were mostly enriched (Fig. 1A). The majority of the X-chromosome DNA products amplified from DOP-PCR were enriched from 200 bp to 1500 bp. DNA products exhibited an even distribution among different sizes (Fig. 1B).

### Cloning and screening DNA genomic subtractive library

From the subtractive library, 2700 single colonies were obtained, among which 480 single colonies with lengths between 250 and 1500 bp were randomly selected for PCR amplification (Suppl. material 1: Fig. S2). To identify Y-specific DNA, the hybridization of spinach female/male genomic DNA with DNA sequences from subtractive library was performed. Unique hybridization between male spinach and DNA could be potentially from Y chromosome. Fifty-five Y chromosome-specific recombinant plasmids in spinach were identified by dot blot hybridization (Suppl. material 1: Fig. S3).

### Screening male-biased repetitive DNA sequences

Sanger sequencing of the 55 selected DNA sequences yielded a total of 30, 576 bp of product, ranging from 248 bp to 1, 354 bp in length (MN830920–MN830942, MN810356–MN810387). A total of 12, 049 bp of DNA products were identified as repeat sequences using RepeatMasker softerware (http://www.repeatmasker.org/), which accounted for 39.4% of the total sequences. Thirty-two of the 55 DNA sequences contained repeat DNA sequences, including *LTR/Copia*, *LTR/Gypsy*, simple repeats and DNA/CMC-EnSpm (Suppl. material 1: Table S2). The sequence alignment of these 55 DNA sequences against the NCBI database (nr database) through BLASTn with default settings generated 55 significant hits. Those hits included spinach BAC clones, uncharacterized mRNA, and the transcription factor MYB80 (LOC110782202) of spinach from previous annotations and BAC libraries (*S. oleracea* L.) (Table 1).

**Table 1.** BLAST search for spinach male-biased DNA fragments.

ID	Accession number	Size	Description	Query Cover	E Value	Per. Ident
SP1-3	MN830920	661	PREDICTED: *Spinacia oleracea* uncharacterized LOC110790287 (LOC110790287), transcript variant X4, ncRNA	96%	0	94.25%
SP1-71	MN830921	326	Select seq AP017640.1*Spinacia oleracea* DNA, BAC clone: 009-126-13E-1, strain: 03-009, complete sequence	96%	3.00E-162	100.00%
SP1-86	MN810356	293	Select seq AP017641.1*Spinacia oleracea* DNA, BAC clone: 009-160-1L-1, strain: 03-009, complete sequence	93%	2.00E-103	92.03%
SP1-89	MN810357	294	Select seq AP017637.1*Spinacia oleracea* DNA, BAC clone: 009-26-14K-1, strain: 03-009, complete sequence	93%	2.00E-137	99.28%
SP2-26	MN810358	248	PREDICTED: *Spinacia oleracea* uncharacterized LOC110800978 (LOC110800978), mRNA	94%	1.00E-109	97.87%
SP3-4	MN810359	270	Select seq XM_021992625.1 PREDICTED: *Spinacia oleracea* uncharacterized LOC110787992 (LOC110787992), mRNA	98%	1.00E-79	87.41%
SP3-8	MN810360	261	*Spinacia oleracea* mitochondrion, complete genome	96%	3.00E-116	97.23%
SP3-36	MN830922	334	PREDICTED: *Spinacia oleracea* uncharacterized LOC110799754 (LOC110799754), mRNA	96%	1.00E-160	99.07%
SP3-88	MN830923	293	*Spinacia oleracea* DNA, BAC clone: 009-26-14K-1, strain: 03-009, complete sequence	68%	3.00E-67	91.04%
SP4-1	MN810361	433	Select seq XM_022007996.1 PREDICTED: *Spinacia oleracea* uncharacterized LOC110802566 (LOC110802566), mRNA	99%	6.00E-168	91.88%
SP4-2	MN810362	432	PREDICTED: *Spinacia oleracea* uncharacterized LOC110791229 (LOC110791229), mRNA	99%	0	95.82%
SP4-3	MN810363	423	PREDICTED: *Spinacia oleracea* uncharacterized LOC110802566 (LOC110802566), mRNA	96%	5.00E-179	94.51%
SP4-4	MN810364	426	Select seq XM_022001236.1 PREDICTED: *Spinacia oleracea* uncharacterized LOC110796204 (LOC110796204), mRNA	98%	0	94.41%
SP4-7	MN810365	432	PREDICTED: *Spinacia oleracea* uncharacterized LOC110802566 (LOC110802566), mRNA	98%	0	96.49%
SP4-8	MN810366	433	PREDICTED: *Spinacia oleracea* uncharacterized LOC110802566 (LOC110802566), mRNA	98%	0	96.73%
SP4-10	MN810367	432	Select seq XM_022007996.1 PREDICTED: *Spinacia oleracea* uncharacterized LOC110802566 (LOC110802566), mRNA	98%	0	96.02%
SP4-11	MN810368	423	PREDICTED: *Spinacia oleracea* uncharacterized LOC110802566 (LOC110802566), mRNA	98%	8.00E-177	93.68%
SP4-38	MN810369	293	*Spinacia oleracea* DNA, BAC clone: 009-123-11N-1, strain: 03-009, complete sequence	97%	1.00E-135	97.90%
SP4-48	MN830924	575	Select seq AP017641.1*Spinacia oleracea* DNA, BAC clone: 009-160-1L-1, strain: 03-009, complete sequence	36%	2.00E-61	88.78%
SP4-53	MN830925	262	PREDICTED: *Spinacia oleracea* probable methyltransferase PMT15 (LOC110782476), mRNA	31%	2.00E-29	97.59%
SP5-1	MN810370	410	Select seq AP017639.1*Spinacia oleracea* DNA, BAC clone: 009-123-11N-1, strain: 03-009, complete sequence	49%	2.00E-87	96.53%
SP5-2	MN830926	365	Select seq AP017641.1*Spinacia oleracea* DNA, BAC clone: 009-160-1L-1, strain: 03-009, complete sequence	56%	1.00E-94	97.61%
SP5-3	MN830927	365	*Spinacia oleracea* DNA, BAC clone: 009-160-1L-1, strain: 03-009, complete sequence	57%	2.00E-93	97.14%
SP5-4	MN830928	356	Select seq AP017639.1*Spinacia oleracea* DNA, BAC clone: 009-123-11N-1, strain: 03-009, complete sequence	56%	4.00E-55	87.13%
SP5-5	MN830929	534	*Spinacia oleracea* DNA, BAC clone: 009-126-13E-1, strain: 03-009, complete sequence	37%	7.00E-88	97.46%
SP5-9	MN830930	430	Select seq XM_022005381.1 PREDICTED: *Spinacia oleracea* uncharacterized LOC110800092 (LOC110800092), mRNA	51%	9.00E-92	95.05%
SP5-10	MN810371	409	*Spinacia oleracea* DNA, BAC clone: 009-123-11N-1, strain: 03-009, complete sequence	50%	3.00E-86	95.63%
SP5-11	MN810372	485	*Spinacia oleracea* DNA, BAC clone: 009-126-13E-1, strain: 03-009, complete sequence	42%	4.00E-90	97.97%
SP5-12	MN830931	326	Select seq AP017640.1*Spinacia oleracea* DNA, BAC clone: 009-126-13E-1, strain: 03-009, complete sequence	96%	1.00E-161	100.00%
SP5-48	MN830932	277	Select seq AP017638.1*Spinacia oleracea* DNA, BAC clone: 009-41-10L-1, strain: 03-009, complete sequence	94%	1.00E-75	87.17%
SP6-20	MN810373	573	Chain A, Cryo-EM structure of the spinach chloroplast ribosome reveals the location of plastid-specific ribosomal proteins and extensions	92%	6.00E-171	87.52%
SP7-3	MN810374	549	Select seq XM_021994667.1 PREDICTED: *Spinacia oleracea* uncharacterized LOC110789945 (LOC110789945), mRNA	79%	6.00E-133	86.73%
SP7-4	MN830933	504	PREDICTED: *Spinacia oleracea* transcription factor MYB80 (LOC110782202), mRNA	95%	0	98.96%
SP7-5	MN830934	587	*Spinacia oleracea* mitochondrion, complete genome	94%	0	98.74%
SP7-7	MN810375	536	PREDICTED: *Spinacia oleracea* uncharacterized LOC110777888 (LOC110777888), mRNA	53%	3.00E-116	93.73%
SP7-9	MN830935	504	Select seq XM_021986329.1 PREDICTED: *Spinacia oleracea* transcription factor MYB80 (LOC110782202), mRNA	93%	0	98.94%
SP7-10	MN810376	536	PREDICTED: *Spinacia oleracea* uncharacterized LOC110783205 (LOC110783205), mRNA	53%	9.00E-107	91.64%
SP7-11	MN830936	563	Select seq XM_021982478.1 PREDICTED: *Spinacia oleracea* pentatricopeptide repeat-containing protein At5g02860 (LOC110777897), mRNA	94%	0	99.06%
SP10-9	MN810377	790	Select seq XM_022003128.1 PREDICTED: *Spinacia oleracea* uncharacterized LOC110797998 (LOC110797998), mRNA	98%	0	84.22%
SP13-1	MN810378	267	Select seq XM_021992625.1 PREDICTED: *Spinacia oleracea* uncharacterized LOC110787992 (LOC110787992), mRNA	97%	2.00E-83	88.97%
SP13-2	MN810379	267	PREDICTED: *Spinacia oleracea* uncharacterized LOC110787992 (LOC110787992), mRNA	97%	2.00E-73	86.69%
SP17-1	MN810380	715	PREDICTED: *Spinacia oleracea* uncharacterized LOC110799950 (LOC110799950), mRNA	91%	0	91.10%
SP17-2	MN830937	1128	Select seq AP017639.1*Spinacia oleracea* DNA, BAC clone: 009-123-11N-1, strain: 03-009, complete sequence	98%	0	93.99%
SP51-1	MN830938	758	PREDICTED: *Spinacia oleracea* tudor domain-containing protein 3 (LOC110774971), transcript variant X2, mRNA	51%	6.00E-178	99.71%
SP51-2	MN830939	766	PREDICTED: *Spinacia oleracea* tudor domain-containing protein 3 (LOC110774971), transcript variant X2, mRNA	45%	1.00E-179	100.00%
SP51-3	MN830940	758	PREDICTED: *Spinacia oleracea* tudor domain-containing protein 3 (LOC110774971), transcript variant X2, mRNA	51%	1.00E-179	100.00%
SP52-1	MN830941	644	Select seq AP017637.1*Spinacia oleracea* DNA, BAC clone: 009-26-14K-1, strain: 03-009, complete sequence	42%	5.00E-79	87.73%
SP52-3	MN830942	638	*Spinacia oleracea* DNA, BAC clone: 009-26-14K-1, strain: 03-009, complete sequence	42%	5.00E-69	85.87%
SP55-1	MN810381	1001	Select seq AP017640.1*Spinacia oleracea* DNA, BAC clone: 009-126-13E-1, strain: 03-009, complete sequence	93%	0	84.31%
SP55-3	MN810382	1001	PREDICTED: *Spinacia oleracea* uncharacterized LOC110802605 (LOC110802605), mRNA	50%	0	99.28%
SP55-4	MN810383	1001	PREDICTED: *Spinacia oleracea* uncharacterized LOC110802605 (LOC110802605), mRNA	53%	0	99.78%
SP73	MN810384	1318	Select seq AP017638.1*Spinacia oleracea* DNA, BAC clone: 009-41-10L-1, strain: 03-009, complete sequence	100%	0	99.85%
SP75	MN810385	1354	*Spinacia oleracea* DNA, BAC clone: 009-41-10L-1, strain: 03-009, complete sequence	100%	0	99.93%
SP76	MN810386	1163	PREDICTED: *Spinacia oleracea* uncharacterized LOC110779482 (LOC110779482), partial mRNA	45%	3.00E-152	85.66%
sp77	MN810387	1154	PREDICTED: *Spinacia oleracea* uncharacterized LOC110779482 (LOC110779482), partial mRNA	45%	3.00E-147	85.23%

### Chromosome localization of repetitive DNA sequences

Using the 32 DNA sequences that contained repeat DNA sequences as probes, we tried to identify the distribution of fluorescence signals on the Y chromosome. However, no fluorescence signals were found on the chromosomes using four simple repeats (SP5-1, SP55-1, SP55-3 and SP55-4) and two DNA/CMC-EnSpm DNA sequences (SP55-3 and SP55-4). When four *LTR/Copia* DNA sequences (SP3-4, SP3-8, SP17-1 and SP1-86) were selected to be used as probes, the signals showed a dispersed distribution in all chromosomes (Suppl. material 1: Figure S4). Four DNA sequences (SP73, SP75, SP76, and SP77) containing *Ty3-gypsy* family RTs were selected as probes for FISH (Suppl. material 1: Table S2).

Four pairs of primers were generated according to the DNA sequences SP73, SP75, SP76, and SP77 (Suppl. material 1: Table S1). PCR and gel electrophoresis assays generated one band for each DNA sample ranging from 1, 000 bp to 2, 000 bp (Suppl. material 1: Fig. S5). Interestingly, one 1318 bp sequence of SP73 shared high percentage identity with Y-specific BAC clone 009-41-10L-1 from previous studies (AP017638.1) (99%) (Suppl. material 1: Fig. S6). Another target band of a 1354 bp-SP75 DNA sequence (Suppl. material 1: Fig. S5) containing 907 bp Gypsy/DIRS1 RT sequence also presented a high-percentage homologous DNA with Y -specific BAC clone 009-41-10L-1 (AP017638.1) (99%) (Suppl. material 1: Fig. S7) and with a male-specific SCAR marker of spinach (FJ169475.1) (99%). SP76 was 1, 163 bp (Suppl. material 1: Fig. S5) and contained 939 bp Gypsy/DIRS1 RT DNA sequence. SP77 was 1, 154 bp (Suppl. material 1: Fig. S5) and contained 940 bp Gypsy/DIRS1 RT DNA sequence. PCR products with specific amplified bands were purified to construct fluorescent probes.

**Figure 2.**
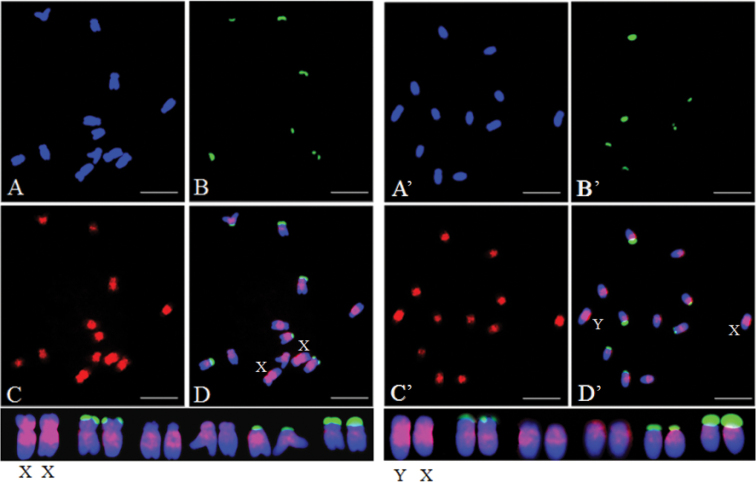
Distribution patterns of hybridization signals from female and male spinach using 45S rDNA (green) and SP73 (red) as probes **A** (**A**’), DAPI **B** (**B**’), 45S rDNA (green) as probe **C** (**C**’), SP73 (red) as probe **D** (**D**’), The merged figure of **A** (**A**’), **B** (**B**’) and **C** (**C**’). Scale bas: 10 μm.

**Figure 3.**
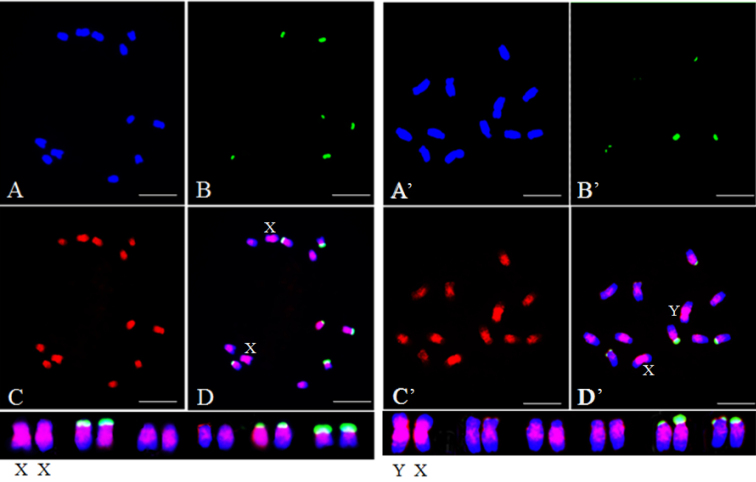
Distribution patterns of hybridization signals from female and male spinach using 45S rDNA (green) and SP75 (red) as probes **A** (**A**’), DAPI **B** (**B**’), 45S rDNA (green) as probe **C** (**C**’), SP75 (red) as probe **D** (**D**’), The merged figure of **A** (**A**’), **B** (**B**’) and **C** (**C**’). Scale bars: 10 μm.

**Figure 4.**
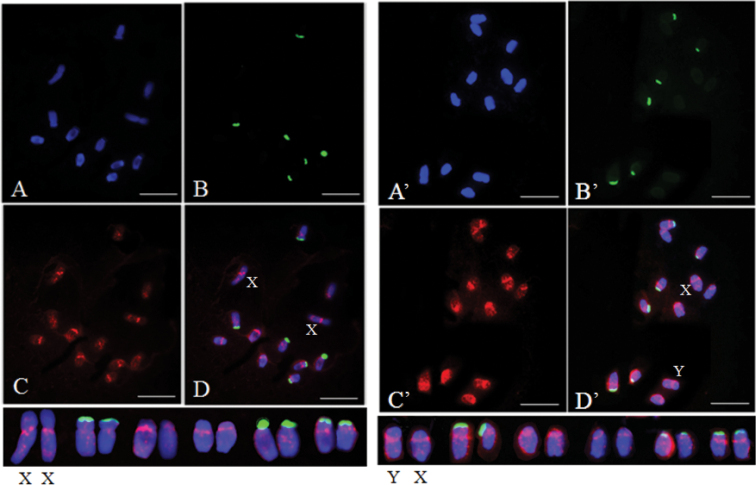
Distribution patterns of hybridization signals from female and male spinach using 45S rDNA (green) and SP76 (red) as probes **A** (**A**’), DAPI **B** (**B**’), 45S rDNA (green) as probe **C** (**C**’), SP76 (red) as probe **D** (**D**’), The merged figure of **A** (**A**’), **B** (**B**’) and **C** (**C**’). Scale bars: 10 μm.

**Figure 5.**
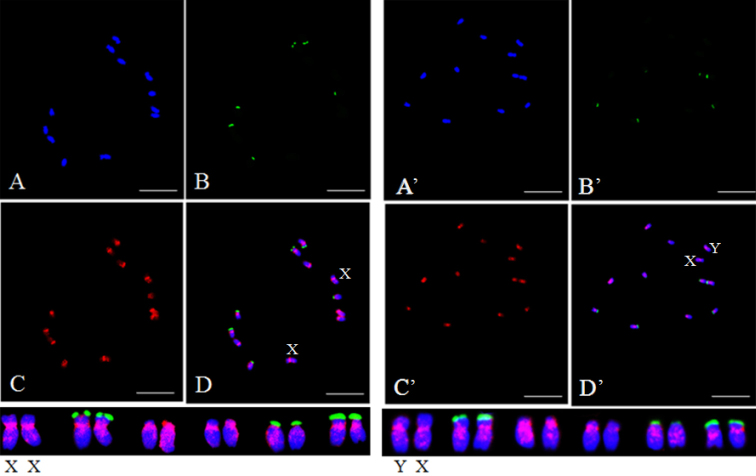
Distribution patterns of hybridization signals from female and male spinach using 45S rDNA (green) and SP77 (red) as probes **A** (**A**’) DAPI **B** (**B**’) 45S rDNA (green) as probe **C** (**C**’) SP77 (red) as probe **D** (**D**’). The merged figure of **A** (**A**’), **B** (**B**’) and **C** (**C**’). Scale bars: 10 μm.

For chromosomal localization, 45S rDNA was used as a probe to distinguish each chromosome, the prominent fluorescent signals of which were observed on chromosomes 2, 5, and 6 (Deng et al. 2012). Initially, the homologous sex chromosome pair was identified by 45S rDNA probes based on the weak signals from X and Y chromosomes (Deng et al. 2012). This was the largest homologous pair among the six pairs of chromosomes. FISH performed using SP73 and SP75 as DNA probes detected an even distribution of fluorescence signal on each pair of homologous chromosomes. Those signals were concentrated near the putative centromere and pericentromeric regions. Stronger signals observed on the sex chromosomes compared to that on the A chromosomes. Nevertheless, no significant difference of fluorescence signals was found between X and Y chromosomes (Figs 2, 3). In addition, hybridization signals primarily appeared near the putative centromeres for each homologous chromosome pair by using SP76 and SP77 probes for FISH, and sporadic signals existed on the long arms. X and Y chromosomes were not distinguished based on signals (Figs 4, 5).

## Discussion

### The establishment of single chromosome subtractive hybridization technique in spinach

Chromosome microdissection technology has the advantage of being able to isolate specific DNA products from a single chromosome. Moreover, the isolated products of the target sequences can be enriched through PCR (Zhou et al. 2007). In this study, we combined the conventional genomic subtraction hybridization with single chromosome microdissection to rapidly clone male-biased DNA sequences from spinach sex chromosomes. Twenty-one of 55 cloned DNA sequences were partially overlapped to BAC clone 009-126-13E-1, BAC clone 009-160-1L-1, BAC clone 009-26-14K-1, BAC clone 009-123-11N-1, and BAC clone 009-41-10L-1 located on the male-determining region of the spinach Y chromosome (Kudoh et al. 2018). Specifically, repetitive DNA sequence SP73 and SP75 shared more than 1000 bp highly-homologous sequences compared with the reported BAC clone 009-41-10L-1. The technique proposed above can be used to screen sex-biased DNA fragments of X and Y chromosomes in spinach.

### FISH localization of male-biased repetitive DNA sequences in spinach

Sex reversal from hermaphroditism to dioecy in flowering plants requires two mutants, namely, one male-sterile mutant (generally for the first time) and one female-sterile mutant. These mutant sites are used to stabilize sex on a pair of chromosomes (Charlesworth and Charlesworth 1978, Charlesworth 2013). Subsequently, suppression recombination occurs and plays a substantial role in maintaining dioecy by preventing the segregation of hermaphrodite or sterile progenies through crossing over. Repetitive sequence insertion plays a major role in ceasing recombination and leads to the formation of sex determination region and divergence of homologous sex chromosomes. A large number of transposon DNA sequences were enriched in the sex-determining region of asparagus (Jamsari et al. 2004). In *S. latifolia*, the Y chromosome is the largest and has accumulated a large number of repeated DNA sequences. Further analysis showed that transposons are inserted into the Y chromosome more frequently than into the other parts of the genome. FISH results showed that *Gypsy*RTs on Y chromosome of *S. latifolia* were 2.7 times more than those of *Copia*RTs (Cermak et al. 2008). Kudoh et al. (2018) reported that a higher amount of repetitive DNA sequences has accumulated near the Y linked region in spinach. However, very few direct cytological studies have explored the repetitive DNA sequences around the sex-determining locus. The BAC clone sequences are only a part of the sex-linked non-recombining region (Kudoh et al. 2018). Thus, other strategies should be used to explore the DNA sequences on a non-recombining region. On the basis of the DOP-PCR products of X and Y chromosomes from previous studies (Deng et al. 2013), genomic subtractive hybridization and dot-blot hybridization identified the specific repetitive DNA sequences on spinach sex chromosomes. In this study, the obtained Y chromosome-specific repetitive DNA sequences are mostly *Ty3-gypsy* family RTs. However, Kudoh et al. (2018) reported that most of the repeats found in the Y chromosome region around the male-determining locus are novel *Ty1-copia-like*RTs. The main reason for this could be the fact that the BAC clone sequences is only part of the sex-linked non-recombining region. Moreover, FISH localization identified the distribution pattern of repetitive DNA sequences on the sex chromosomes and autosome of spinach. The fluorescence signals of the DNA sequences containing *LTR/Copia* were distributed in a dispersed manner on all chromosomes (Suppl. material 1: Fig. S4). Although the localization of SP73, SP75, SP76 and SP77 did not differ between sex and autosomes, it did differ on the concentration of signals. It is evident that sex chromosomes have an accumulation of such repeats (SP73 and SP75). However, no significant difference was found in fluorescence signal intensity between X and Y chromosomes (Figs 2–5). The limited discrepancy of repetitive DNA detected between X and Y chromosome could be related to the early stages of sex chromosomes in spinach. Another reason for this result maybe is that dot blotting tends to produce false positives. In *Spinacia*, except for the cultivated spinach accession, *S. turkestanica* (Iljin, 1936) and *S. tetrandra* (Steven ex M. Bieb., 1808) are two extra species. Heteromorphic sex chromosomes were found in some accessions from *S. tetrandra* besides the common homomorphic sex chromosomes (Fujito et al. 2015). Thus, male-biased repetitive DNA sequences obtained in this study can be used as probes to explore their distribution in the homomorphic and heteromorphic sex chromosomes and to elucidate their possible role in the evolution of spinach sex chromosome.

## Supplementary materials

Supplementary material 1 Figures S1–S6, Table S1 Data type Figure/Table Explanation note **Figure S1.** Procedure of isolation of biggest chromosome in spinach by micromanipulator. **Figure S2.** Partial PCR products of recombinant clones using M13R and M13F as primers. **Figure S3.** 3 Dot blot hybridization results of partial subtractive hybridization clones. **Figure S4.** Distribution patterns of hybridization signals from female and male spinach using 45S rDNA (green) and SP1-86 (red) as probes. **Figure S5.** PCR amplification result of SP73, SP75, SP76 and SP77 marker. **Figure S6.** Pair-wise alignment between SP73 and BAC clone 009-41-10L-1. **Figure S7.** Pair-wise alignment between SP75 and BAC clone 009-41-10L-1. **Table S1.** Primer sequences for repetitive DNA sequences. **Table S2.** Summary of repetitive elements in 55 DNA Sequences. https://binary.pensoft.net/file/534966This dataset is made available under the Open Database License (http://opendatacommons.org/licenses/odbl/1.0/). The Open Database License (ODbL) is a license agreement intended to allow users to freely share, modify, and use this Dataset while maintaining this same freedom for others, provided that the original source and author(s) are credited.Jian Zhou, Shaojing Wang, Li’ang Yu, Ning Li, Shufen Li, Yulan Zhang, Ruiyun Qin, Wujun Gao, Chuanliang Deng
