# Supplementary material for: Cloning and physical localization of male-biased repetitive DNA sequences in Spinacia oleracea (Amaranthaceae)
Source: Comp Cytogenet. 2021 Apr 23;15(2):101–18. doi: 10.3897/CompCytogen.v15i2.63061 (PMC8087614; doi:10.3897/CompCytogen.v15i2.63061)
Supplement: Supplementary material 1 — Figures S1–S6, Table S1 [file comparative_cytogenetics-15-101-s001.docx]

**Supplementary Materials for**

**Cloning and physical localization of male-biased repetitive DNA sequences in spinach**

Jian Zhou1**, Shaojing Wang1**, Li'ang Yu2, Ning Li1, Shufen Li1, Yulan Zhang1, Ruiyun Qin1, Wujun Gao1, Chuanliang Deng1*

^1^ College of Life Sciences, Henan Normal University, Xinxiang 453007, China,

^2^ Department of Plant Biology, University of Illinois at Urbana-Champaign, Urbana, IL 61801, USA

*Correspondence: [dengchuanliang@htu.cn](mailto:dengchuanliang@htu.cn)

**Co-first author


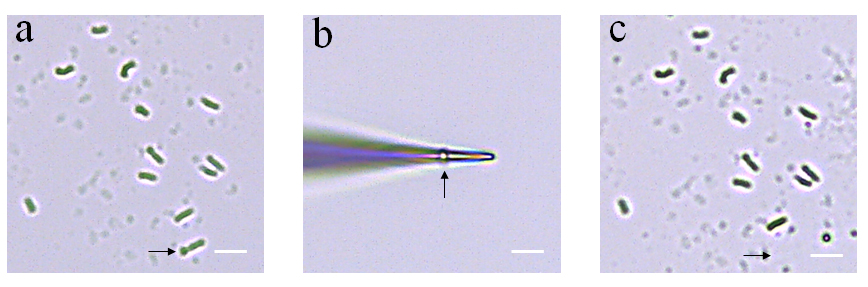


**Supplemental Figure 1 Procedure of isolation of biggest chromosome in spinach by micromanipulator.** a Mitosis metaphase chromosomes of a root tip cell before chromosome isolation (Arrow showed the biggest chromosome), b the target chromosome adhering to the tip of a glass needle, c metaphase chromosomes after isolation of the chromosome indicated by arrow in a by a glass needle. Bar 10 μm


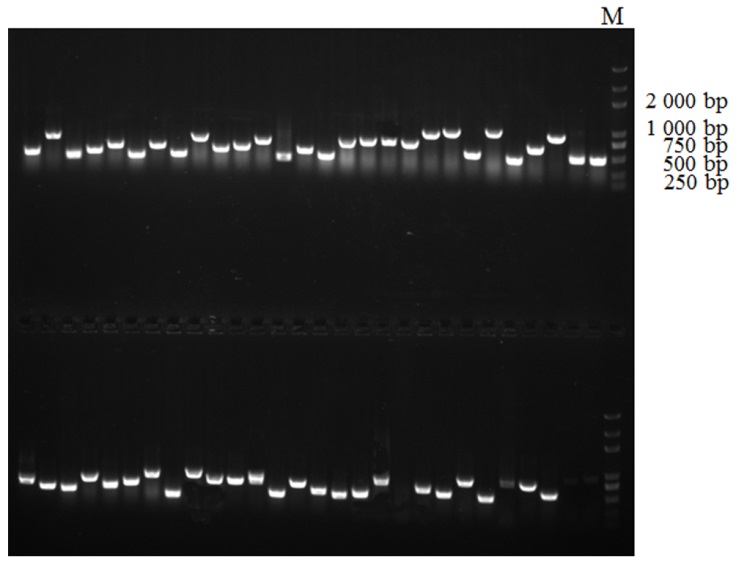
**Supplemental Figure 2 Partial PCR products of recombinant clones using M13R and M13F as primers.** M: Trans 2K Plus DNA Marker.


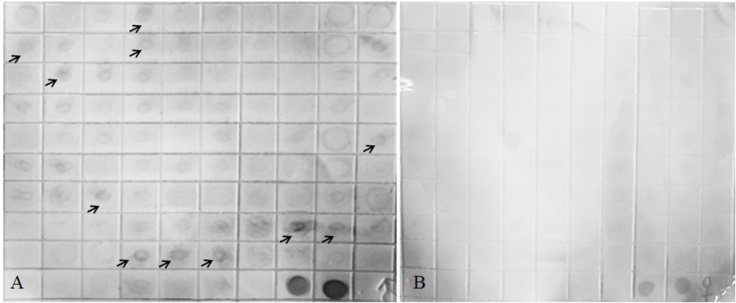
**Supplemental Figure 3 Dot blot hybridization results of partial subtractive hybridization clones.** Dot blot hybridization with DIG-labeled the genome DNA of male spinach (A) and the genome DNA of female spinach (B); Arrows showed male biased dots.


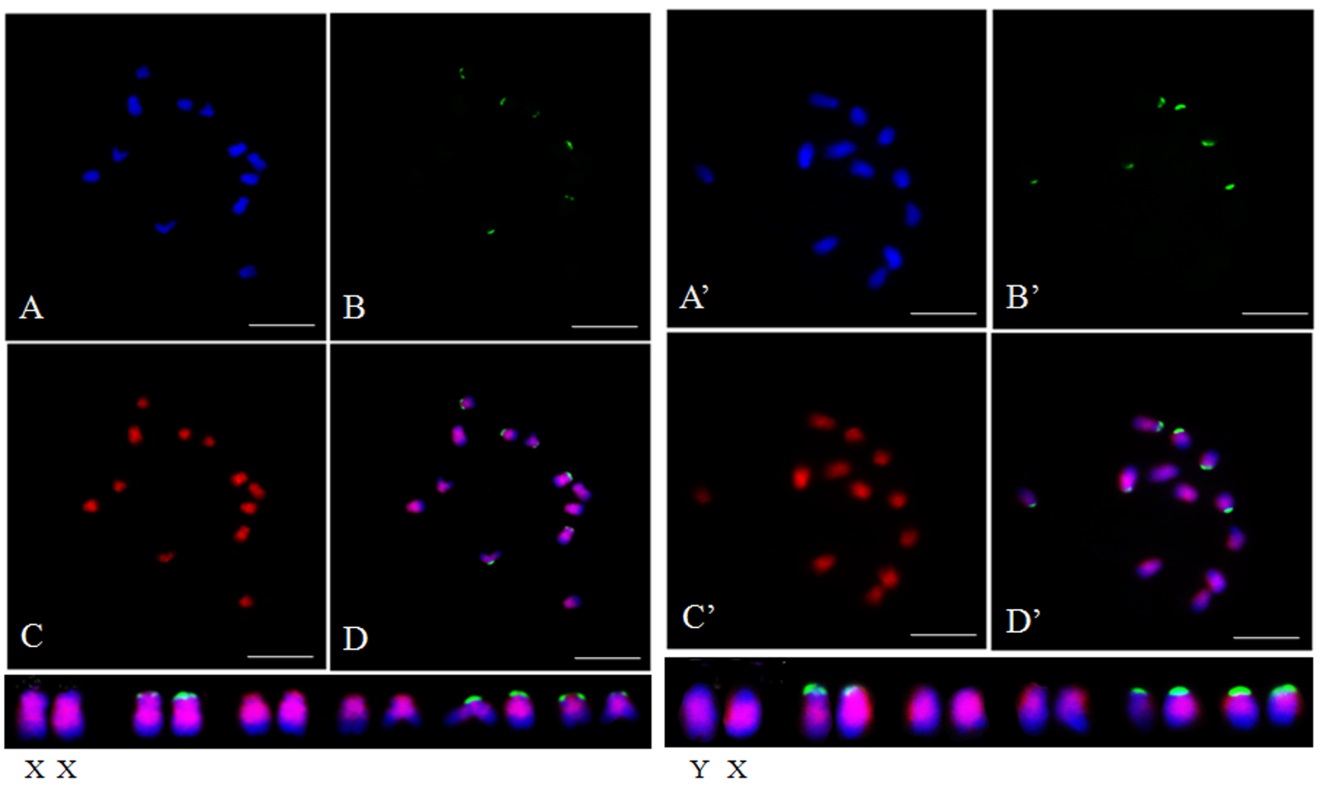
**Supplemental Figure 4 Distribution patterns of hybridization signals from female and male spinach using 45S rDNA (green) and SP1-86 (red) as probes.** A (A’), DAPI; B (B’), 45S rDNA (green) as probe; C (C’), SP1-86 (red) as probe; D (D’), The merged figure of A (A’), B (B’) and C (C’). (Bar=10 μm)


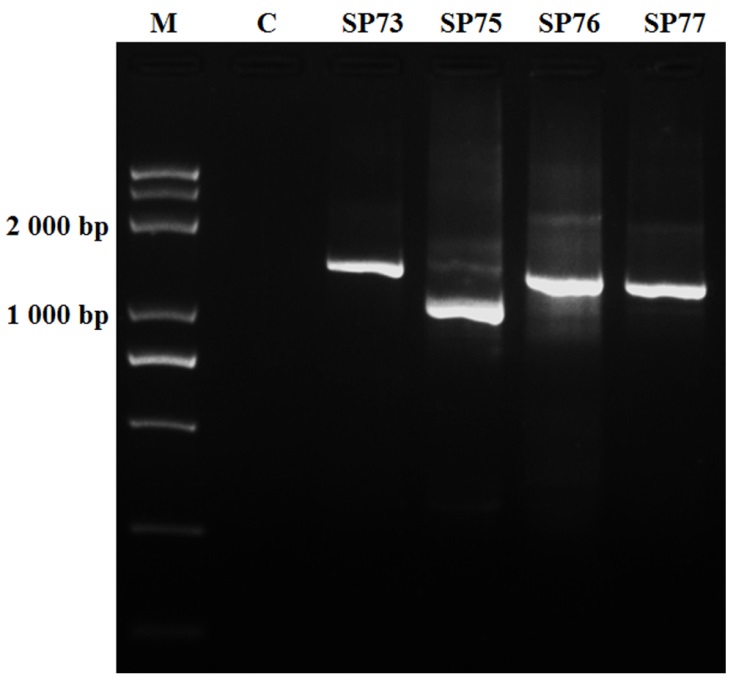
**Supplemental Figure 5 PCR amplification result of SP73, SP75, SP76 and SP77 marker.** M, Ttrans 2K Plus DNA Marker; C, negative control.


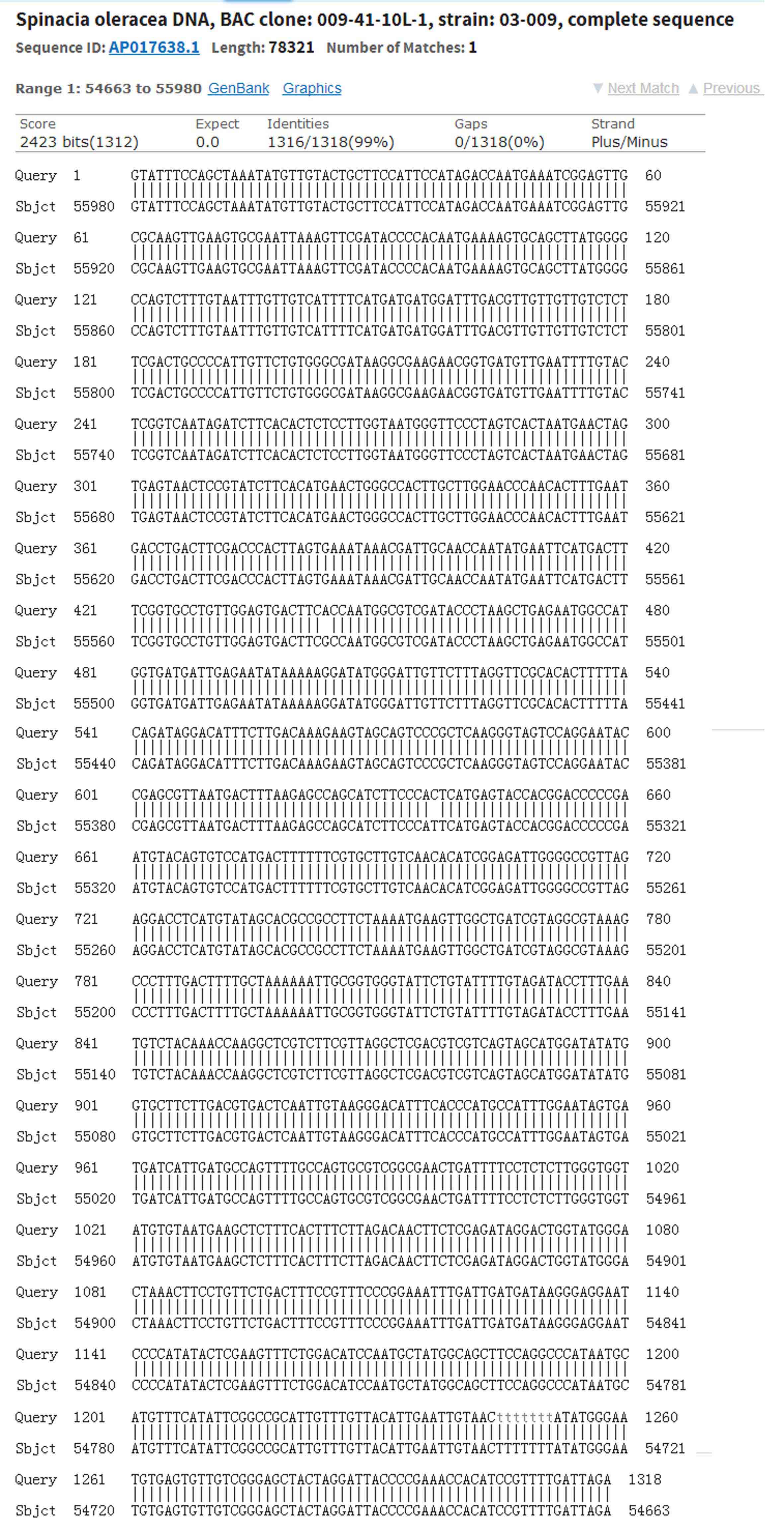


**Supplemental Figure** **6 Pair-wise alignment between SP73 and BAC clone 009-41-10L-1.**


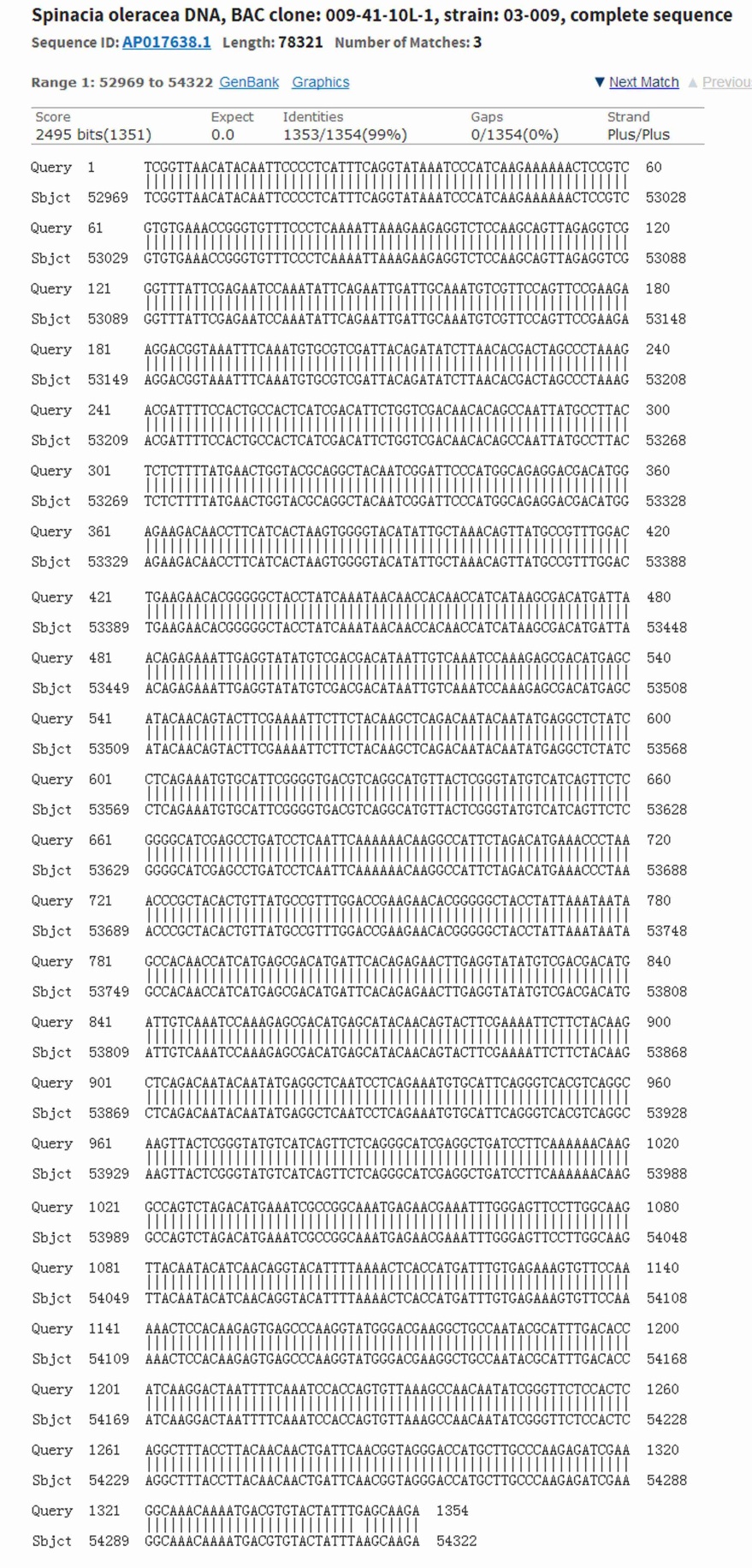


**Supplemental Figure 7 Pair-wise alignment between SP75 and BAC clone 009-41-10L-1.**

**Supplemental Table 1 Primer sequences for repetitive DNA sequences.**

| Primer | 5’→3’ | Annealing temperature |
| --- | --- | --- |
| 73-F | GTATTTCCAGCTAAATATGTTGT | 51℃ |
| 73-R | CTAATCAAAACGGATGTGGT | 51℃ |
| 75-F | CTTGCTCAAATAGTACACGTC | 54℃ |
| 75-R | TCGGTTAACATACAATTCCC | 51℃ |
| 76-F | ATTTCAAAACACGATGCTC | 52℃ |
| 76-R | TAGAGGCCATATATAGACTCA | 52℃ |
| 77-F | TATACTGGCATCTGTATTCCC | 52℃ |
| 77-R | TGGTATTAAATACAGACAACCGA | 54℃ |
